# The low-dose colchicine in patients after non-CABG cardiac surgery: a randomized controlled trial

**DOI:** 10.1186/s13054-023-04341-9

**Published:** 2023-02-07

**Authors:** Tuo Pan, Chen-Yu Jiang, He Zhang, Xi-Kun Han, Hai-Tao Zhang, Xin-Yi Jiang, Wei Chen, Kuo Wang, Fu-Dong Fan, Jun Pan, Qing Zhou, Chuang-Shi Wang, Li Zhang, Dong-Jin Wang

**Affiliations:** 1 Department of Cardio-Thoracic Surgery, Nanjing Drum Tower Hospital, Peking Union Medical College and Chinese Academy of Medical Sciences, Graduate School of Peking Union Medical College, Number 321 Zhongshan Road, Nanjing, 210008 Jiangsu China; 2 Department of Cardio-Thoracic Surgery, Nanjing Drum Tower Hospital, The Affiliated Hospital of Nanjing University Medical School, Nanjing, China; 3 Department of Cardio-Thoracic Surgery, Shanghai Children’s Medical Center, School of Medicine, Shanghai Jiao Tong University, Shanghai, China; 4 Department of Epidemiology, Harvard University T H Chan School of Public Health, Boston, MA USA; 5 Program in Genetic Epidemiology and Statistical Genetics, Harvard University T H Chan School of Public Health, Boston, MA USA; 6 Department of Cardio-Thoracic Surgery, Nanjing Drum Tower Hospital, Nanjing Medical University, Nanjing, China; 7 Department of Cardio-Thoracic Surgery, Nanjing Drum Tower Hospital, The Affiliated Clinical College of Xuzhou Medical University, Nanjing, China; 8 Medical Research and Biometrics Center, National Center for Cardiovascular Diseases, Fuwai Hospital, Chinese Academy of Medical Sciences and Peking Union Medical College, Mentougou District, Beijing, 102300 China; 9 Hongqiao International Institute of Medicine, Tongren Hospital, Shanghai Jiao Tong University School of Medicine, Shanghai, 200336 China

**Keywords:** Colchicine, Cardiopulmonary bypass, Myocardial injury, Troponin T, Cardiovascular surgery

## Abstract

**Background:**

Recent high-quality trials have shown that the anti-inflammatory effects of colchicine reduce the risk of cardiovascular events in patients suffering post-myocardial infarction and chronic coronary disease. The effect of colchicine in patients undergoing non-coronary artery bypass grafting (non-CABG) with cardiopulmonary bypass remains unclear. We aim to evaluate the effect of colchicine on myocardial protection in patients who underwent non-CABG cardiac surgery.

**Method:**

Patients were randomly assigned to colchicine or placebo groups starting 72 h before scheduled cardiac surgery and for 5 days thereafter (0.5 mg daily).The primary outcome was the level of cardiac troponin T (cTnT) at postoperative 48 h. The secondary outcomes included troponin I (cTnI) and creatine kinase-MB (CK-MB), inflammatory biomarkers (procalcitonin and interleukin-6, etc.), and adverse events (30-day mortality, stroke, ECMO and IABP use, etc.).

**Results:**

A total of 132 patients underwent non-CAGB cardiac surgery, 11were excluded because of diarrhea (*n* = 6) and long aortic cross-clamp time > 2 h (*n* = 5), 59 were assigned to the colchicine group and 62 to the placebo group. Compared with the placebo group, cTnT (median: 0.3 μg/L, IQR 0.2–0.4 μg/L vs. median: 0.4 μg/L, IQR 0.3–0.6 μg/L, *P* < 0.01), cardiac troponin I (median: 0.9 ng/ml, IQR 0.4–1.7 ng/ml vs. median: 1.3 ng/ml, IQR 0.6–2.3 ng/ml, *P* = 0.02), CK-MB (median: 1.9 ng/ml, IQR 0.7–3.2 ng/ml vs. median: 4.4 ng/ml, IQR 1.5–8.2 ng/ml, *P* < 0.01), and interleukin-6 (median: 73.5 pg/ml, IQR 49.6–125.8 pg/ml vs. median: 101 pg/ml, IQR 57.5–164.7 pg/ml, *P* = 0.048) were significantly reduced in colchicine group at postoperative 48 h. For safety evaluation, the colchicine (*n* = 65) significantly decreased post-pericardiotomy syndrome (3.08% vs. 17.7%, *P* < 0.01) and increased the rate of diarrhea (9.23% vs. 0, *P* = 0.01) compared with the placebo group (*n* = 62). No significant difference was observed in other adverse events between the two groups.

**Conclusion:**

A short perioperative course of low-dose colchicine was effective to attenuate the postoperative biomarkers of myocardial injury and inflammation, and to decrease the postoperative syndrome compared with the placebo.

*Trial registration *ChiCTR2000040129. Registered 22nd Nov. 2020. This trial was registered before the first participant was enrolled. http://www.chictr.org.cn/showproj.aspx?proj=64370.

## Background

Over the past years, several substantial clinical trials have accumulated identifying inflammatory processes as key mediators of the deleterious inflammatory processes as myocardial ischemia–reperfusion-related phenomena in patients presenting with myocardial infarction [1], post-coronary artery bypass grafting (post-CABG) [2], and post-percutaneous coronary intervention (post-PCI) [3]. The central role of inflammation in the progression of coronary arterial disease (CAD) is well recognized [4, 5]. Colchicine, as a traditional type of anti-inflammatory drug, is widely applied to treat gout, familial Mediterranean fever, and pericarditis [6]. Its mechanism is not fully understood but includes reduced responsiveness of neutrophil adhesion [7], and a suppressed activation of the neutrophils extracellular traps (NETs) which participates in myocardial injury [8]. In the colchicine cardiovascular outcomes trial (COLCOT) study, colchicine led to a significantly lower risk of ischemic cardiovascular events than placebo [9]. A high-quality clinical study also showed the potential benefit of colchicine in ST-segment-elevation myocardial infarction [1]. It is clear that colchicine can play an important role in cardioprotection in patients with CAD.

Cardiopulmonary bypass (CPB) is a necessary life support during open-heart surgery. Inflammatory response caused by many factors including CPB has been well known to increase postoperative morbidity and mortality [10, 11]. Inflammation-induced myocardial ischemia–reperfusion injury is frequently observed in patients after cardiac surgery and significantly affects prognosis despite the application of cardioplegic solution during cardiac operation [12, 13]. Therefore, the colchicine which is an oral anti-inflammatory drug may have cardioprotective effects in patients with CPB. A previous randomized control trial (RCT) has demonstrated that colchicine might reduce CABG-related myocardial injury [2]. However, there was lack of data regarding the effect of colchicine administrated in patients who undergo valvular, aortic, or congenital heart surgery. Our study, therefore, aims to evaluate the effect of low-dose colchicine on myocardial protection in patients who undergo non-CABG cardiac surgery.

## Methods

### Study population

This study is an investigator-initiated, single center, single-blind, randomized, placebo-controlled clinical trial. It was initiated in November 2020 and completed in August 2022 after receiving approval from the ethical committee of Nanjing Drum Tower Hospital (2020-293-02) and registered in the Chinese Clinical Trial Registry (ChiCTR2000040129). The study enrolled 132 patients with CPB from the department of cardiothoracic in Nanjing Drum Tower Hospital. Written informed consent was obtained from all patients before enrollment. Patients were randomized in a 1:1 ratio to receive either colchicine 0.5 mg once daily or placebo for 3 days before the operation and for 5 days after operation. The design of the trial has been published previously [14]. The database from this study was approved to be shared by Nanjing Drum Tower Hospital.

The inclusion criteria were as follows: Adult patients undergoing on-pump cardiovascular surgery, aged 50–75 (including 50 and 75), male or female, have signed the informed consent form (ICF); patients with New York Heart Association Class (NYHA): I-II.

The “low-risk” patients were enrolled in our study based on following exclusion criteria: patients undergoing emergency surgery; patients undergoing deep hypothermic circulatory arrest surgery; patients with atrial fibrillation who need radiofrequency catheter ablation; with coronary artery disease who need percutaneous transluminal coronary intervention or CABG; poor hepatorenal dysfunction (Child Pugh Class B or C, estimated glomerular filtration rate < 35 mL/min/1.73 m^2^); baseline inflammatory indicators abnormal [interleukin-6 (IL-6) > 10 pg/mL, procalcitonin (PCT) > 0.5 ng/mL, C-reactive protein (CRP) > 10 mg/L]; predictive mortality of European System for Cardiac Operative Risk Evaluation (EuroSCORE II) [15] > 3%; had received cardiac surgery; diagnosed with inflammatory immune diseases; received treatment of colchicine or hormone previously; had a history of tumor or infectious disease; patients who had colchicine allergy or intolerance; and needed ventricular outflow reconstruction in the surgery. Patients who had gastrointestinal upset would refuse to continuously administrate colchicine in our study (*n* = 6). And the long aortic cross-clamp (ACC) time (> 120 min) is usually predicted severe myocardial injury in our hospital [16]. Therefore, patients would be removed after randomization when the intraoperative ACC times > 120 min.

### Blinding and randomization

Patients, outcome assessors, and statisticians will be blinded. Patients in the colchicine treatment group will be given colchicine tablets, and those in the control group will be given starch tablets. We will remove the name of medicine packaging so that patients will not know which group they are in. A research assistant who will not be involved in the study intervention and evaluation will be in charge of the randomization. The random numbers will be generated using Microsoft Excel software in a block size of 4. Patients will be enrolled in a ratio of 1:1. Each patient will get a number according to the date when they sign the informed consent form.

### Safety evaluation and postoperative outcomes

In some centers, the cardiac troponin I (cTnI) is daily tested after cardiac surgery to diagnose myocardial injury. However, the cTnT is routinely tested in our hospital, and the cTnI was tested by self-purchasing kit (Xintong Medical Technology Co., LTD, Suzhou, China). Some studies had reported that cardiac troponin T (cTnT) at the postoperative 48 h impacted survival after cardiac surgery [12, 17, 18]^.^ And, to avoid potential bias from our self-tested cTnI, the primary outcome in our study was the level of cTnT at postoperative 48 h. The secondary outcomes were measured as follows: other biomarkers of myocardial injury, including creatine kinase-MB (CK-MB), cTnI, myohemoglobin (MYO), b-type natriuretic peptide (BNP), and D-dimer; inflammatory biomarkers, including white blood cells (WBC), c-reactive protein (CRP), interleukin-6(IL-6), and procalcitonin (PCT); and the adverse outcomes, including 30-days mortality mechanical ventilation time > 48 h, re-operative, stroke, extracorporeal membrane oxygenation (ECMO), and continuous renal replacement therapy (CRRT), post-pericardiotomy syndrome (PPS), etc. [14, 19]. The PPS includes: fever lasting beyond the first postoperative week without evidence of systemic or focal infection; pleuritic chest pain; friction rub; evidence of pleural effusion; and evidence of new or worsening pericardial effusion [19].

### Medical intervention

All patients underwent cardiac surgery applying standard protocols by Prof. Dong-Jin Wang. The ascending aorta was cannulated with a patient size-appropriate cannula. Venous cannulations were chosen with separate cannulas in the superior and inferior vena cava. Based on an active clotting time of more than 480 s, heparin (200–400 U/kg) was used to achieve anticoagulation. Systemic temperature was kept in a range of 32–34 °C. The CPB circuit was primed with 1500–2000 ml of sodium acetate Ringer’s injection, 10–30 g of albumin, and 2.5 g of magnesium sulfate injection (concentration: 10%). The initial volume of the antegrade cold blood cardioplegia solution (4:1 ratio) was needed for the cessation of all cardiac electrical activity but never less than 20 ml/kg. Cardiac arrest was maintained, after 30 min of initiated antegrade infusion, by the retrograde infusion of 10 ml/kg of blood cardioplegia solution every 15 min. The antegrade strategy would be following implemented if retrograde infusion had been used 3 times. All patients were transferred to the intensive care unit (ICU) after surgery and then were extubated within postoperative 24 h. Patients in two groups would be given colchicine or placebo for the first postoperative days after tracheal extubation. Blood samples for cTnT, cTnI, CK-MB, MYO, BNP measurement and other markers were obtained at admission and then on the first postoperative day (POD1), the second postoperative day (POD2), the third postoperative day (POD3), the fourth postoperative day (POD4), and the fifth postoperative day (POD5).

### Statistical analysis

The sample size was calculated by PASS (V.11) software. This study is a parallel randomized controlled study, and the primary outcome is the level of cTnT on POD2. Previous measurements in patients after cardiac surgery in our institution had an average cTnT level of 0.5 ± 0.25 μg/L on POD2. With 60 patients in each group undergoing randomization after screening, we estimated that the trial would have a 30% reduction in the cTnT with 90% power (type II error probability 0.1), and at a two-sided *α*-level (type I error probability) of 0.05. Considering a dropout rate of 10%, 66 patients would be required in each group. It is acceptable that 121 enrolled patients (59 vs. 62) in our final study sample.

The IBM SPSS statistical software (Statistics for Windows, version 25, IBM Corporation, Armonk, NY, USA) and R software (Version: 4.2.2) were used for analysis. Continuous variables were presented as the mean ± SD or, if appropriate, as the median with interquartile ranges (IQR). Discrete variables are depicted as frequencies (*n*, %). Normally distributed continuous variables were evaluated using Student’s *t* test, or the Mann–Whitney U nonparametric method was used for non-normally distributed continuous variables. Continuous variables were determined to be normally distributed by the Shapiro–Wilk test. Categorical data were compared using the chi-square test or Fisher’s exact test. Differences of biomarkers from POD1 to POD5 between the two groups were also analyzed by repeated-measures ANOVA or nonparametric analysis for repeated measurements as appropriated. *P* value of < 0.05 was considered statistically significant.

## Results

**Fig. 1:**
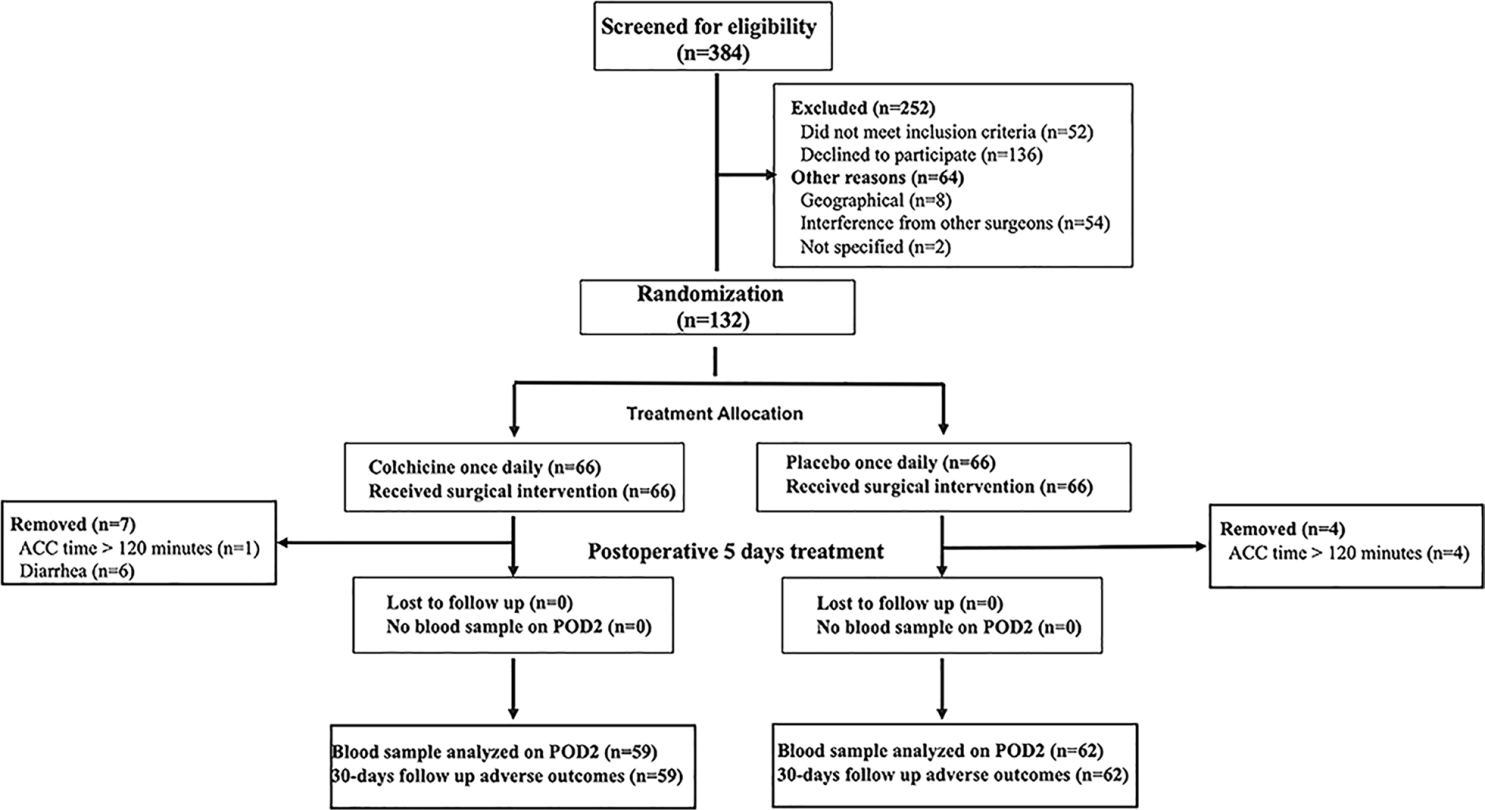
Study flowchart. ACC: aortic cross-clamp, POD2: the second postoperative day

Among the 384 patients who had potential eligibility to participate (Fig. 1), 132 underwent randomization and 121 received at least one dose of colchicine (*n* = 59) or placebo (*n* = 62). Among the 24 patients (15.4%) who had signed the informed consent form but did not undergo randomization, the reason was sudden unexpected rejection by patients or patients’ family members. Among 132 randomized patients, 11 patients were removed because of diarrhea (6: 0 = colchicine/placebo, 4.5%) and long aortic cross-clamp time > 2 h (1:4 = colchicine: placebo, 3.8%).

The baseline characteristics of the patients were well balanced between the two groups (Table 1). The mean (± SD) age of the patients was 59.4 ± 6.1 years, and 57.8% were males. New York Heart Association (NYHA) class (*P* = 0.9), EuroSCORE II, type of cardiac surgery, CPB time, and aortic cross-clamp (ACC) time had no difference between the colchicine group and placebo group. For safety evaluation, the postoperative outcomes were acceptable (Table 2). All of the patients in the two groups were extubated within postoperative 24 h. Compared with the placebo group, the colchicine significantly decreased PPS (*P* < 0.01), pleuritic chest pain (*P* < 0.05), friction rub (*P* < 0.05), and pleural effusion (*P* < 0.01). In electrocardiogram (ECG) monitoring, compared with the placebo group, the colchicine group showed a non-significantly lower rate of ST-elevation (*P* = 0.07). No significant difference was observed in other adverse outcomes between the two groups though colchicine increased the rate of diarrhea (9.23% vs. 0, *P* = 0.01).

**Table 1:** Characteristics of the trial patients at baseline

Characteristic	Total (*n* = 121)	Colchicine (*n* = 59)	Placebo (*n *= 62)
Age (year)	59.42 ± 6.11	58.90 ± 6.15	59.92 ± 6.08
Gender (male, %)	70, 57.85%	31, 52.54%	39, 62.90%
Weight (kg)	65.64 ± 10.75	66.86 ± 11.26	64.48 ± 10.19
Body surface area (m^2^)	1.69 ± 0.17	1.71 ± 0.18	1.68 ± 0.17
Body mass index (kg/m^2^)	24.01 ± 2.70	24.42 ± 2.89	23.63 ± 2.47
*NYHA class (n, %)*			
I	64, 52.89%	31, 52.54%	33, 53.22%
II	57, 47.11%	28, 47.46%	29, 46.77%
EuroSCORE II (%)	1.80(1.71–1.98)	1.79(1.68–1.93)	1.91 ± 0.36
Preoperative LVEF (%)	55.00(45.00–58.00)	55.00(50.00–58.00)	55.00(42.5–57.00)
*Medical history (n,%)*			
Diabetes Mellitus	13, 10.74%	7, 11.86%	6, 9.68%
Hypertension	53, 43.80%	30, 50.85%	23, 37.10%
Chronic kidney diseases	5, 4.13%	2, 3.34%	3, 4.84%
Chronic lung diseases	0	0	0
Stroke	8, 6.61%	4, 6.78%	4, 6.45%
Marfan syndrome	0	0	0
Peripheral arterial disease	2, 1.65%	1, 1.69%	1, 1.61%
Smoking	63, 52.07%	28, 47.46%	35, 56.45%
Drinking	57, 47.11%	27, 45.76%	30, 48.38%
Previous non-cardiac operation	2, 1.65%	1, 1.69%	1, 1.61%
*Type of cardiac operation (n,%)*			
Mitral valve replacement	35, 28.92%	17, 28.82%	18, 29.03%
Aortic valve replacement	32, 26.46%	15, 25.42%	17, 27.42%
Mitral + aortic valve replacement	26, 21.48%	12, 20.33%	14, 22.58%
Atrial septal defect repair	5, 4.11%	4, 6.78%	1, 1.62%
Bentall/Wheat’s/Ascending aorta replacement	16,13.22%	8,13.57%	8, 12.90%
Atrial myxoma resection	7, 5.78%	3, 5.08%	4, 6.45%
CPB time (minutes)	110.53 ± 31.52	113.24 ± 28.99	107.95 ± 33.79
ACC time (minutes)	75.0(56.0–97.0)	81.0(58.0–97.0)	69.0(55.0–97.50)
*Medication use (n,%)*			
*β*-blocker	40, 33.06%	22, 37.29%	18, 29.03%
ACEi/ARB	51, 42.15%	27, 45.76%	23, 37.10%
Calcium channel blocker	34, 28.10%	17, 28.81%	17, 27.42%
Diuretic	31, 25.62%	16, 27.12%	15, 24.19%

*NYHA* New York Heart Association, *CPB* Cardiopulmonary bypass, *ACEi* Angiotensin-converting enzyme inhibitor

**Table 2:** Safety evaluation

Variable	Colchicine (*n* = 65)*	Placebo (*n* = 62)		*P* value
*Adverse outcomes (n, %)*				
30-day mortality	0	0		–
MV > 48 h	0	0		–
Cardiac re-operation	0	0		–
ECMO use	0	0		–
IABP use	0	0		–
Stroke	0	0		–
CRRT use	0	0		–
*PPS within POD14 (n, %)*	2, 3.08%	11, 17.74%		0.006
Fever beyond first postoperative week	1, 1.54%	4, 6.45%		0.155
Pleuritic chest pain	0	4, 6.45%		0.037
Friction rub	1, 1.54%	7,11.29%		0.030
Pleural effusion	1, 1.54%	11, 17.74%		0.002
New or worsening pericardial effusion	0	0		–
*Other outcomes (n, %)*				
Infection	2, 3.08%	3, 4.84%		0.610
Cardiac tamponade	0	0		–
Constrictive pericarditis	0	0		–
*ECG monitoring in ICU (n, %)*				
Hyperacute T-wave	2, 3.08%	6, 9.68%		0.126
ST-elevation	4, 6.16%	10, 16.13%		0.073
Left/right bundle branch block	2, 3.08%	6, 9.68%		0.126
Atrial fibrillation	1, 1.54%	2, 3.23%		0.531
Other ventricular arrhythmias	2, 3.08%	6, 9.68%		0.126
Gastrointestinal disturbances (*n*, %)	6, 9.23%	0		0.014
Length of ICU stay (days)	3.0 (2.00–3.0)	2.0 (2.0–3.0)		0.112
Length of hospital stay (days)	15.0 (14.0–18.5)	16.0 (14.0–17.2)		0.692
MV time (hours)	6.0 (4.5–7.5)	7.0 (4.0–10.1)		0.156

*MV* Mechanical ventilation, *CRRT* Continuous renal replacement therapy, *Median* (Interquartile range), *ECMO* Extracorporeal membrane oxygenation, *IABP* Intra-aortic balloon pump, *PPS* Post-pericardiotomy syndrome, *ECG* Electrocardiogram, *POD14* Postoperative 14 days

*6 patients who occurred gastrointestinal disturbances (diarrhea) before surgery and refused to continuously administrate colchicine in the colchicine group were included in the safety analysis

**Fig. 2:**
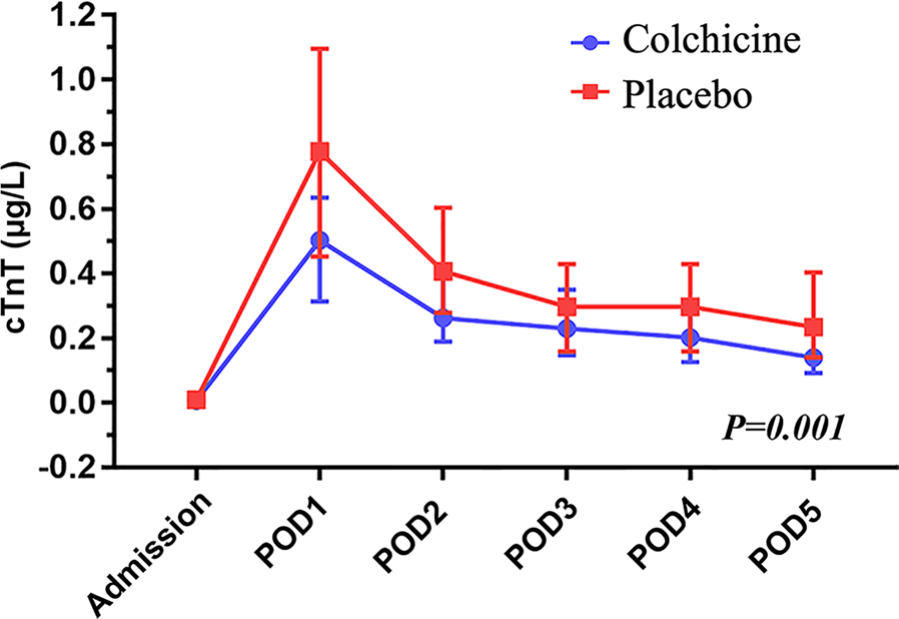
The preoperative and postoperative cardiac troponin T (cTnT)

**Fig. 3:**
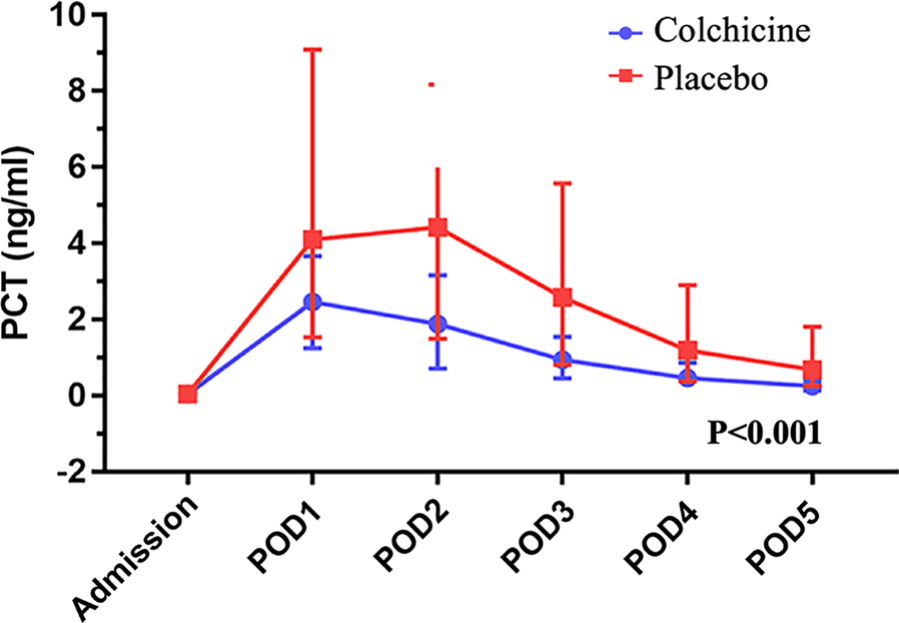
The preoperative and postoperative procalcitonin (PCT)

**Fig. 4:**
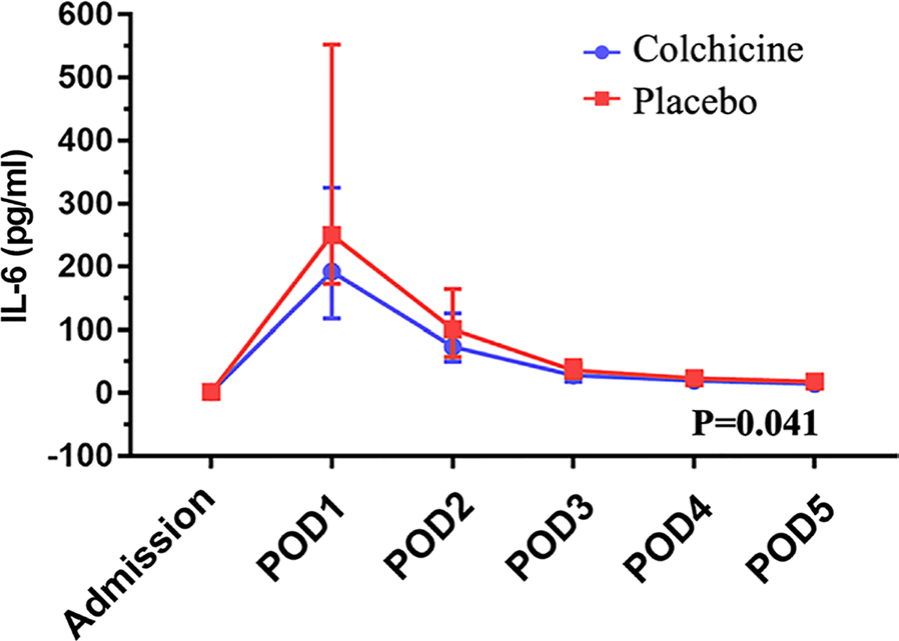
The preoperative and postoperative interleukin-6 (IL-6)

The baseline (at admission) of blood tests was a well balance between the two groups (Table 3). Compared with the placebo group, the colchicine group showed a significantly decreased level of cTnT (*P* < 0.01), cTnI (*P* = 0.02), CK-MB (*P* = 0.01), IL-6 (*P* < 0.05), and PCT (*P* < 0.01) on the POD2. When analyzing by taking repeated measurements into account, the colchicine group still had lower levels of cTnT (Fig. 2, *P* < 0.01), PCT (Fig. 3, *P* < 0.01) and IL-6 (Fig. 4, *P* < 0.05) and had a non-significantly lower level of cTnI (*P* = 0.06) within postoperative 5 days compared with the placebo group. The detailed data are presented in Table 3.

**Table 3:** Blood results within postoperative 5 days

Variable	Colchicine (*n* = 59)	Placebo (*n* = 62)	*P* value
*cTnT (μg/L)*			0.001*
Admission (*n*_1_:*n*_0_ = 59: 62)	0.007 (0.006–0.01)	0.009 (0.007–0.01)	0.131
POD 1 (*n*_1_:*n*_0_ = 59: 62)	0.50 (0.36–0.63)	0.82 (0.52–1.14)	< 0.001
POD 2 (*n*_1_:*n*_0_ = 59: 62)	0.27 (0.21–0.41)	0.41 (0.30–0.60)	0.003
POD 3 (*n*_1_:*n*_0_ = 59: 62)	0.23 (0.15–0.35)	0.35 (0.23–0.49)	0.005
POD 4 (*n*_1_:*n*_0_ = 59: 59)	0.20 (0.13–0.30)	0.30 (0.17–0.43)	0.009
POD 5 (*n*_1_:*n*_0_ = 57: 57)	0.14 (0.09–0.21)	0.23 (0.14–0.40)	0.001
*cTnI (ng/ml)*			0.062*
Admission (*n*_1_:*n*_0_ = 59: 62)	0.03 (0.02–0.03)	0.03 (0.03–0.03)	0.532
POD 1 (*n*_1_:*n*_0_ = 59: 61)	1.88 (1.00–3.44)	3.43 (1.37–5.81)	0.014
POD 2 (*n*_1_:*n*_0_ = 59: 61)	0.88 (0.43–1.69)	1.25 (0.57–2.37)	0.016
POD 3 (*n*_1_:*n*_0_ = 57: 61)	0.52 (0.23–0.83)	0.67 (0.29–1.26)	0.009
POD 4 (*n*_1_:*n*_0_ = 58: 60)	0.29 (0.13–0.50)	0.48 (0.19–0.79)	0.013
POD 5 (*n*_1_:*n*_0_ = 50: 59)	0.21 (0.10–0.37)	0.34 (0.12–0.58)	0.039
*CK-MB (ng/ml)*			0.494*
Admission (*n*_1_:*n*_0_ = 59: 61)	1.23 (1.00–1.53)	1.30 (1.00–1.73)	0.101
POD 1 (*n*_1_:*n*_0_ = 59: 61)	20.97 (11.49–32.90)	25.97 (17.03–39.72)	0.069
POD 2 (*n*_1_:*n*_0_ = 59: 61)	5.01 (1.79–8.78)	8.91 (3.12–12.60)	0.011
POD 3 (*n*_1_:*n*_0_ = 55: 61)	2.05 (1.37–2.98)	2.54 (1.60–3.75)	0.734
POD 4 (*n*_1_:*n*_0_ = 52: 59)	1.37 (1.00–1.90)	1.52 (1.10–2.20)	0.812
POD 5 (*n*_1_:*n*_0_ = 44: 55)	1.10 (1.00–1.41)	1.17 (1.00–1.75)	0.347
*Myohemoglobin (ng/ml)*			0.594*
Admission (*n*_1_:*n*_0_ = 55: 62)	12.90 (10.58–16.38)	13.26 (10.53–17.24)	0.430
POD 1 (*n*_1_:*n*_0_ = 59: 61)	93.49 (52.55–120.73)	88.49 (49.70–134.65)	0.447
POD 2 (*n*_1_:*n*_0_ = 59: 61)	33.74 (25.00–54.89)	38.05 (27.57–57.90)	0.222
POD 3 (*n*_1_:*n*_0_ = 57: 61)	19.73 (13.49–31.09)	21.78 (15.52–31.20)	0.183
POD 4 (*n*_1_:*n*_0_ = 58: 60)	16.23 (12.86–24.08)	16.75 (12.89–24.64)	0.722
POD 5 (*n*_1_:*n*_0_ = 51: 59)	14.90 (11.27–21.15)	16.45 (12.25–20.62)	0.527
*B-Type natriuretic peptide (pg/ml)*			0.326*
Admission (*n*_1_:*n*_0_ = 59: 62)	48.00 (25.60–132.75)	81.66 (26.82–182.00)	0.057
POD 1 (*n*_1_:*n*_0_ = 59: 61)	439.00 (237.00–769.00)	470.50 (244.00–755.00)	0.773
POD 2 (*n*_1_:*n*_0_ = 59: 61)	582.00 (327.60–853.00)	512.50 (201.00–861.00)	0.544
POD 3 (*n*_1_:*n*_0_ = 57: 61)	491.00 (328.00–747.00)	420.30 (217.00–823.00)	0.316
POD 4 (*n*_1_:*n*_0_ = 57: 60)	389.00 (226.80–604.00)	310.00 (190.00–620.00)	0.574
POD 5 (*n*_1_:*n*_0_ = 51: 59)	351.00 (247.00–482.00)	275.50 (146.00–578.55)	0.241
*White cell count (*× *10*^*9*^*/L)*			**0.133**
Admission (*n*_1_:*n*_0_ = 59: 62)	5.43 ± 1.41	5.73 ± 2.21	0.375
POD 1 (*n*_1_:*n*_0_ = 59: 62)	11.79 ± 4.05	12.47 ± 3.58	0.326
POD 2 (*n*_1_:*n*_0_ = 59: 62)	12.22 ± 3.57	12.86 ± 3.42	0.318
POD 3 (*n*_1_:*n*_0_ = 59: 62)	9.10 ± 2.51	10.19 ± 3.35	0.047
POD 4 (*n*_1_:*n*_0_ = 59: 59)	7.23 ± 2.15	7.78 ± 2.09	0.163
POD 5 (*n*_1_:*n*_0_ = 57: 60)	6.94 ± 2.28	7.50 ± 2.09	0.169
*Neutrophil (%)*			**0.156**
Admission (*n*_1_:*n*_0_ = 59: 62)	57.29 ± 7.23	59.06 ± 10.56	0.282
POD 1 (*n*_1_:*n*_0_ = 59: 62)	88.62 ± 3.25	88.41 ± 3.04	0.711
POD 2 (*n*_1_:*n*_0_ = 59: 62)	86.27 ± 3.73	87.08 ± 2.88	0.186
POD 3 (*n*_1_:*n*_0_ = 59: 62)	81.69 ± 5.39	82.71 ± 3.92	0.234
POD 4 (*n*_1_:*n*_0_ = 59: 59)	75.01 ± 6.12	76.72 ± 5.30	0.106
POD 5 (*n*_1_:*n*_0_ = 57: 60)	70.10 ± 6.77	70.71 ± 6.23	0.612
*Serum creatinine (μmol/L)*			0.161*
Admission (*n*_1_:*n*_0_ = 59: 62)	62.90 (55.50–72.00)	65.00 (55.00–75.00)	0.432
POD 1 (*n*_1_:*n*_0_ = 59: 62)	58.50 (52.00–69.00)	64.00 (56.00–75.00)	0.220
POD 2 (*n*_1_:*n*_0_ = 59: 62)	60.00 (49.50–72.00)	68.00 (59.00–78.00)	0.161
POD 3 (*n*_1_:*n*_0_ = 59: 62)	60.50 (53.00–74.50)	68.00 (58.00–86.00)	0.085
POD 4 (*n*_1_:*n*_0_ = 55: 60)	58.50 (50.50–76.00)	63.00 (55.00–78.00)	0.444
POD 5 (*n*_1_:*n*_0_ = 52: 60)	59.00 (50.00–72.00)	63.00 (53.00–74.00)	0.103
*IgA (g/L)*			0.537*
Admission (*n*_1_:*n*_0_ = 56: 61)	2.07 (1.76–2.44)	2.02 (1.42–2.69)	0.412
POD 3 (*n*_1_:*n*_0_ = 55: 58)	1.47 (1.30–1.81)	1.41 (1.16–1.91)	0.326
POD 5 (*n*_1_:*n*_0_ = 48: 56)	1.75 (1.52–1.97)	1.70 (1.37–2.31)	0.804
*IgG (g/L)*			0.679*
Admission (*n*_1_:*n*_0_ = 56: 61)	11.72 ± 2.39	10.84 ± 2.95	0.079
POD 3 (*n*_1_:*n*_0_ = 55: 58)	7.48 ± 1.57	7.23 ± 1.83	0.428
POD 5 (*n*_1_:*n*_0_ = 48: 56)	8.11 ± 1.56	8.30 ± 2.24	0.603
*IgM (g/L)*			0.058*
Admission (*n*_1_:*n*_0_ = 56: 61)	1.02 (0.71–1.68)	0.86 (0.64–1.16)	0.092
POD 3 (*n*_1_:*n*_0_ = 55: 58)	0.66 (0.48–0.96)	0.57 (0.42–0.71)	0.091
POD 5 (*n*_1_:*n*_0_ = 48: 56)	0.88 (0.74–1.26)	0.78 (0.59–1.16)	0.280
*IgE (IU/ml)*			0.628*
Admission (*n*_1_:*n*_0_ = 56: 61)	46.00 (17.25–114.75)	38.01 (14.50–146.50)	0.545
POD 3 (*n*_1_:*n*_0_ = 55: 58)	50.00 (21.00–164.00)	45.01 (18.75–149.25)	0.934
POD 5 (*n*_1_:*n*_0_ = 48: 56)	63.50 (26.00–222.50)	59.50 (24.00–272.75)	0.976
*Complement-C3 (g/L)*			0.411*
Admission (*n*_1_:*n*_0_ = 56: 61)	1.08 (0.98–1.20)	1.09 (0.94–1.15)	0.060
POD 3 (*n*_1_:*n*_0_ = 55: 58)	1.01 ± 0.21	0.94 ± 0.19	0.074
POD 5 (*n*_1_:*n*_0_ = 48: 56)	1.15 (1.00–1.41)	1.16 (1.00–1.32)	0.868
*Complement-C4 (g/L)*			**0.638**
Admission (*n*_1_:*n*_0_ = 56: 61)	0.24 ± 0.06	0.22 ± 0.06	0.257
POD 3 (*n*_1_:*n*_0_ = 55: 58)	0.22 ± 0.07	0.22 ± 0.06	0.912
POD 5 (*n*_1_:*n*_0_ = 48: 56)	0.26 ± 0.09	0.27 ± 0.08	0.534
*ESR (mm/h)*			0.832*
Admission (*n*_1_:*n*_0_ = 53: 60)	9 (4–15.5)	9 (3–17)	0.968
POD 1 (*n*_1_:*n*_0_ = 59: 61)	35 (19.5–51.5)	31 (17–49.5)	0.565
POD 2 (*n*_1_:*n*_0_ = 59: 61)	48 (36.5–63)	44 (33.5–64)	0.625
POD 3 (*n*_1_:*n*_0_ = 59: 61)	51 (40–72.5)	51 (38–67)	0.407
POD 4 (*n*_1_:*n*_0_ = 58: 55)	51 (37.5–67.5)	50 (35–66.5)	0.241
POD 5 (*n*_1_:*n*_0_ = 52: 57)	44 (29.5–64.5)	45 (32–64.5)	0.604
*D-Dimer (mg/L)*			0.611*
Admission (*n*_1_:*n*_0_ = 55: 62)	0.23 (0.12–0.41)	0.22 (0.09–0.72)	0.823
POD 1 (*n*_1_:*n*_0_ = 59: 61)	1.31 (0.79–2.53)	1.65 (0.89–2.47)	0.451
POD 2 (*n*_1_:*n*_0_ = 59: 61)	0.84 (0.55–1.43)	0.94 (0.58–1.42)	0.830
POD 3 (*n*_1_:*n*_0_ = 57: 61)	1.58 (1.11–2.73)	1.53 (1.19–3.13)	0.844
POD 4 (*n*_1_:*n*_0_ = 58: 60)	3.01 (2.11–5.31)	3.43 (2.01–5.30)	0.735
POD 5 (*n*_1_:*n*_0_ = 51: 59)	4.14 (2.89–5.98)	4.38 (3.17–6.59)	0.565
*C-reactive protein (mg/L)*			0.337*
Admission (*n*_1_:*n*_0_ = 58: 62)	0.61 (0.35–1.04)	0.61 (0.24–1.62)	0.960
POD 1 (*n*_1_:*n*_0_ = 58: 61)	54.65 (39.94–89.97)	42.20 (36.37–55.65)	0.014
POD 2 (*n*_1_:*n*_0_ = 56: 61)	140.52 (104.40–174.07)	132.56 (96.85–158.37)	0.105
POD 3 (*n*_1_:*n*_0_ = 56: 61)	110.50 (70.53–144.49)	96.10 (73.22–122.68)	0.359
POD 4 (*n*_1_:*n*_0_ = 58: 60)	62.25 (40.75–83.36)	54.51 (39.23–70.88)	0.132
POD 5 (*n*_1_:*n*_0_ = 51: 59)	38.31 (31.70–56.67)	40.04 (30.10–51.60)	0.547
*Interleukin-6 (pg/ml)*			0.041*
Admission (*n*_1_:*n*_0_ = 59: 62)	1.50 (1.50–2.10)	1.50 (1.50–2.08)	0.903
POD 1 (*n*_1_:*n*_0_ = 59: 61)	192.00 (118.01–325.02)	250.21 (172.87–552.29)	0.049
POD 2 (*n*_1_:*n*_0_ = 59: 61)	73.45 (49.64–125.82)	101.00 (57.46–164.69)	0.048
POD 3 (*n*_1_:*n*_0_ = 57: 61)	27.79 (18.12–43.27)	36.40 (23.70–51.72)	0.050
POD 4 (*n*_1_:*n*_0_ = 58: 60)	19.62 (13.44–25.89)	24.34 (17.44–31.67)	0.035
POD 5 (*n*_1_:*n*_0_ = 51: 59)	14.86 (11.89–20.03)	18.00 (10.72–27.50)	0.085
*Procalcitonin (ng/ml)*			< 0.001*
Admission (*n*_1_:*n*_0_ = 59: 62)	0.04 (0.04–0.04)	0.04 (0.04–0.04)	0.406
POD 1 (*n*_1_:*n*_0_ = 59: 61)	2.46 (1.24–3.66)	4.10 (1.53–9.09)	0.008
POD 2 (*n*_1_:*n*_0_ = 59: 61)	1.88 (0.72–3.16)	4.42 (1.49–8.17)	0.001
POD 3 (*n*_1_:*n*_0_ = 57: 61)	0.94 (0.455–1.54)	2.58 (0.81–5.57)	< 0.001
POD 4 (*n*_1_:*n*_0_ = 58: 60)	0.47 (0.24–0.87)	1.19 (0.37–2.90)	< 0.001
POD 5 (*n*_1_:*n*_0_ = 50: 57)	0.25 (0.13–0.42)	0.68 (0.25–1.81)	< 0.001

Mean ± SD or median (first to third quartile)

*For each biomarker, the repeated-measures analysis (repeated-measures ANOVA or nonparametric analysis of repeated measurements) is performed, and the *p* value is presented in the first row

*POD1* The first postoperative day, *POD2* The second postoperative day, *POD3* The third postoperative day, *POD4* The fourth postoperative day, *POD5* The fifth postoperative day, *ESR* Erythrocyte sedimentation rate, *cTnT* Cardiac troponin T, *cTnI* Cardiac troponin I

## Discussion

Among patients with CPB, 0.5 mg of colchicine once daily resulted in lower levels of cTnT, cTnI, and CK-MB on the second postoperative days than the placebo. During the period of postoperative 5 days, the colchicine could also significantly decrease levels of cTnT, IL-6, and PCT. And the level of cTnI might be decreased in the colchicine group during the postoperative 5 days compared with the placebo group. The postoperative troponin levels have been shown to be associated with prognosis [18, 20]. IL-6 and PCT were well-known inflammatory biomarkers, which might be associated with myocardial injury [21, 22]. In this context, these results suggest a beneficial effect of colchicine on myocardial protection in patients undergoing non-CABG cardiac surgery. For the clinical endpoint, colchicine could decrease the PPS. However, major adverse events had no differences between the two groups though colchicine increased the rate of diarrhea. Our finding, which needs to be confirmed in a larger sample study to assess clinical endpoints, suggests a potential role of colchicine in the alleviation of postoperative myocardial injury.

There is great interest in using anti-inflammatory therapies in order to reduce the risk of myocardial ischemia/reperfusion injury. Colchicine deemed as an economical treatment that targets inflammatory mechanisms and seems safe and useful for patients with coronary heart disease [1, 9]. In the COLCOT study which enrolled 4745 patients recruited within 30 days after strike of myocardial infarction, colchicine at a dose of 0.5 mg daily presents a significantly lower risk of ischemic cardiovascular event than placebo [9]. The other low-dose colchicine (LoDoCo2) study showed that colchicine could lower down the risk of cardiovascular events [23]. Colchicine appears to be efficacious and well tolerated for recurrent pericarditis, post-pericardiotomy syndrome, and recurrence of post-procedural atrial fibrillation [19, 24]. According to these high-quality trials, there is no doubt that colchicine could exert cardioprotection through the anti-inflammatory pathway. Meanwhile, in experimental animal models of myocardial inflammatory injury, colchicine has been shown a cardioprotective effect [25]. The anti-inflammatory mechanisms of colchicine are tangled without full understanding but include reduced responsiveness of neutrophils to inflammatory signals [7, 8, 26]. In our study, the inflammatory biomarkers (PCT and IL-6) and myocardial injury biomarkers (cTnT and cTnI) were decreased in the colchicine group. It strengthened the evidence for the conclusion that anti-inflammatory therapies could reduce the severity of myocardial ischemia/reperfusion injury. However, MYO, BNP, D-dimer, WBC count, CRP, and neutrophil count had no differences between the two groups (Table 3). These variables have lower sensitivity to detect inflammatory and myocardial injury than PCT, IL-6, cTnT, and cTnI [27–29], which may be the reason why these biomarkers had no differences between the two groups. Other inflammatory and immunological biomarkers (Table 3), including IgA, IgE, IgM IgG, IgE, complement-C3, complement-C4, and erythrocyte sedimentation rate (ESR), showed no differences before and after surgery between colchicine and placebo, which denied the effects of colchicine on these biomarkers.

The incidence of atrial fibrillation (AF) after cardiac surgery is usually 23.7% [30]. However, in our study, the incidence of postoperative AF (POAF) is 2.36% (3/127). It is an ultra-low incidence of POAF. Nowadays, we have improved our intraoperative clinical practice for preventing POAF. In this study, all of the patients received an intraoperative intervention which was an ablation of the major atrial ganglionic plexi via injection of calcium chloride (CaCl_2_) [31]. Wang et al. [31] reported that CaCl_2_ injection was effective and reduced the incidence of POAF. Owing to CaCl_2_ injection, the incidence of POAF is decreased to 6.89% in patients with acute myocardial infarction-related ventricular septal rupture [32]. Moreover, our study found that colchicine could decrease PPS. It is similar to the COPPS trial. In the COPPS trial, the authors reported that colchicine was safe and efficacious in the prevention of PPS [19]. The PPS has a troublesome course and complicates the postoperative period with even life-threatening events such as cardiac tamponade; prolonged hospital stay; and increased management costs [19]. Therefore, colchicine might represent a primary prevented drug for PPS.

To the best knowledge, our study is the first single-center RCT in the world to assess the effect of colchicine on perioperative cardioprotection in patients who need valvular or aortic surgery but CABG. Giannopoulos et al. [2] reported a cohort of 59 patients who underwent on-pump CABG, and they found that a short perioperative course of colchicine was effective in attenuating postoperative increases in hypersensitive troponin T and CK-MB compared with placebo, while it still needs to consider the factors including the number of arterial or venous bypass grafts during a period of CABG procedure. More importantly, the infarction size usually fluctuates in a broad range, which includes other potential biases that could not be controlled if the patients with CABG procedure were enrolled in the trial. The coronary angiogram before cardiac surgery is routinely implemented in our hospital when patients are more than 50 years old. There were zero patients who had coronary disease in this trial. Acute global ischemia/reperfusion injury is mainly induced by aortic cross-clamping and declamping [2]. Hence, in our study, baseline characteristics were well balanced, the CPB time and ACC time between the colchicine group and the placebo group were similar, and ACC time should be less than 120 min. It ensured the maximized homogeneity between the two groups. In a word, the results of our study are reliable, meaningful, and useful. The colchicine is well tolerated and helpful to reduce myocardial injury in patients with CPB.

### Study limitation

Our study has some limitations. Firstly, this study is a single-blind, randomized, placebo-controlled clinical trial. The single-blind design may affect patients’ selection. And the randomization is not performed by an independent system; the block size is small. These factors may also cause bias. However, to minimize bias as much as possible, we have taken some measures like using objective primary outcome, and doctors are blind to the randomization scheme and personnel training. Secondly, based on our inclusion and exclusion criteria, this study recruited “low-risk” patients. However, the effect of colchicine on myocardial protection in “high-risk” patients was not examined yet. Thirdly, we utilized cTnT to evaluate the perioperative myocardial infarction. In other words, whether colchicine could improve clinical outcomes needs further confirmation. Even though cTnT has been proven to be a well-validated biomarker for postoperative outcome [18], and our findings suggest that colchicine could decrease the incidence of PPS, it still does not equate to other adverse clinical endpoints. It is indeed that a larger sample study is needed to estimate the effects of colchicine on adverse clinical endpoints. Fourthly, the schedule of 0.5 mg colchicine, which remains to be explored in the Chinese population, may not be appropriate for western people. Some inflammatory biomarkers, including IL-10, IL-8, and tumor necrosis factor-1β, might be useful to predict myocardial ischemia–reperfusion injury. However, these biomarkers are not tested in our hospital. Fifthly, according to the previous report, 3 preoperative days and 5 postoperative days are enough to investigate the differences in biomarkers [2]. However, the prolonged duration might result in significant differences in adverse endpoints if the colchicine was used for more than one month. Finally, there were 6 patients who had gastrointestinal disturbances (diarrhea) in the colchicine group. These 6 patients (5 on the second day of taking colchicine and 1 on the first day) occurred diarrhea before cardiac surgery and refused to continuously use the colchicine. Their biomarkers, such as cTnT, cTnI, and CM-MB, did not record after that. Although we included these patients in the safety analysis, the effect of colchicine on biomarkers of myocardial injury and inflammation cannot be reported in this study. These limitations may cause some potential inaccuracies in our study.

## Conclusion

Our study suggests a beneficial effect of colchicine on myocardial protection in patients undergoing non-CABG cardiac surgery. No significant difference was observed in other adverse outcomes between the two groups though colchicine increased the rate of diarrhea. Our finding, which needs to be confirmed in a larger sample study to assess clinical endpoints, suggests a potential role of colchicine in the alleviation of postoperative myocardial injury.

## Data Availability

The data underlying this article are available in [Clinical Trial Management Platform] at http://www.medresman.org.cn/pub/cn/proj/projectshshow.aspx?proj=4760.

## Abbreviations

CPB: Cardiopulmonary bypass
cTnT: Cardiac troponin T
cTnI: Cardiac troponin I
PCT: Procalcitonin
IL-6: Interleukin-6
ACC: Aortic cross-clamp
POD: Postoperative day
CABG: Coronary artery bypass grafting
CK-MB: Creatine kinase-MB
MYO: Myohemoglobin
BNP: B-type natriuretic peptide
PPS: Post-pericardiotomy syndrome
